# Impact of low testing numbers on chronic wasting disease apparent prevalence

**DOI:** 10.1080/19336896.2025.2530534

**Published:** 2025-07-10

**Authors:** Jameson J. Mori, Nelda A. Rivera, William M. Brown, Daniel J. Skinner, Peter E. Schlichting, Jan E. Novakofski, Nohra E. Mateus-Pinilla

**Affiliations:** a Illinois Natural History Survey, Prairie Research Institute, University of Illinois Urbana-Champaign, Champaign, IL, USA; b Illinois Department of Natural Resources, Division of Wildlife Resources, Springfield, IL, USA; c Department of Animal Sciences, University of Illinois Urbana-Champaign, Champaign, IL, USA; d Department of Pathobiology, University of Illinois Urbana-Champaign, Urbana, IL, USA; e Department of Natural Resources & Environmental Sciences, University of Illinois Urbana-Champaign, Urbana, IL, USA

**Keywords:** apparent prevalence, cervid, chronic wasting disease, CWD, diagnostic testing, disease surveillance, wildlife management

## Abstract

Chronic wasting disease (CWD) is a fatal, neurodegenerative disease of cervids, and its management heavily relies on diagnostic testing. Test results are commonly used to calculate ‘apparent prevalence’ (AP) – the percent of animals tested for CWD (CWD tests) with CWD-positive test results (CWD cases) – but this obscures how tests and cases individually contribute to this statistic. This is most relevant when CWD testing is limited because when few animals are tested, detection of even a single infected deer can result in a high AP that poorly reflects reality. We hypothesized that when CWD testing is limited, AP is negatively driven by testing – rather than cases – with more tests corresponding to lower APs. Graphed CWD surveillance data from townships in Illinois and Wisconsin, USA, indicate that CWD AP values ≥50% were only observed when <23 deer were tested. We used Bayesian multilevel zero-inflated Beta regression to model AP as a function of CWD tests, CWD cases and nonlinear transformations of these two terms separately for each state. The best-fit models of both identified a statistically significant negative relationship between AP and testing numbers that was modified by a positive nonlinear test covariate. This means adding tests when testing is low can have a big impact on decreasing the AP, but this relationship weakens as testing increases. We urge treating apparent prevalences ≥50% with caution and emphasize the importance of increasing the test results when initial surveillance has yielded <23 tests.

Chronic wasting disease (CWD) is a fatal neurodegenerative disease that impacts the family *Cervidae* in North America and Europe [1,2]. CWD is of great concern to wildlife managers because it is highly transmissible, 100% lethal, and continues to spread geographically despite efforts to control it [3]. In the United States, CWD is primarily managed at the state level with some assistance from the federal government [4]. Management strategies utilize active and/or passive surveillance, such as testing hunter harvested deer (active) or deer involved in vehicle accidents (passive). In the state of Illinois, recreational hunters provide the majority of CWD samples (99.04%), followed by locally focused culling conducted by the Illinois DNR (0.54%) [1,5,6] and other miscellaneous sources like roadkill.

The data collected during these surveillance efforts can be analysed to prioritize management areas, evaluate the effectiveness of interventions and gain insight into disease dynamics. One essential statistic is *prevalence*, which quantifies the amount of disease present in a certain population during a specified time interval. True prevalence is defined as the number of animals in a population with a disease, divided by the number of animals in that population [7]. When dealing with wildlife, the population – and therefore the true prevalence – is imperfectly known but can be approximated by the apparent prevalence (AP), which is the number of animals that test positive for the disease divided by the total number of animals tested [7]. True prevalence can then be estimated using AP and diagnostic test sensitivity and specificity as inputs to Equation 1 [8].

(1) $$\mathrm{true}\;\mathrm{prevalence}=\frac{\mathrm{apparent}\;\mathrm{prevalence}+\left(\mathrm{specificity}-1\right)}{\mathrm{specificity}+\left(\mathrm{sensitivity}-1\right)}$$

The diagnostic tests used for CWD are enzyme-linked immunosorbent assays (ELISA) and immunohistochemistry (IHC), with the former often used as an initial screening to identify potential cases and the latter used to confirm positive animals [9,10]. AP can be calculated at different spatiotemporal scales and takes values between 0% (none of the animals tested had CWD) and 100% (all animals tested had CWD).

Under ideal conditions, apparent prevalence should equal the true prevalence, but in reality, there are usually biases in the data – such as hunter preference for older males [11,12] - or limitations to the overall sample size that make the AP an imperfect approximation. Investigations of sample size and CWD surveillance have focused on developing better surveillance methods using tools like agent-based modelling [13,14], weighted surveillance strategies [15,16], or Bayesian modelling techniques [17], while research on prevalence itself usually addresses factors that may contribute to observed patterns in wild herds, such as deer age and sex [18]. These studies acknowledge that AP’s inherent dependence on the number of animals tested raises the possibility of AP reflecting sampling effort rather than disease presence and propose future changes to surveillance methods. However, no guidance is offered for assessing and interpreting small datasets that have been, or will be, collected using strategies other than those proposed in the literature.

The problem posed by sample size is a well-documented concern in statistics. The central limit theorem dictates that as sample size increases, statistical estimates based on the sample converge on the true parameter values of the total population. This means that wildlife managers should aim to collect as much data as possible, but at what point is a dataset sufficient for the reliable estimation of CWD apparent prevalence? If the data are not sufficient, how should they be used for management decisions and communicating about the threat of CWD? The answers to these questions have serious implications. Lack of knowledge about adequate sample sizes in CWD surveillance can result in undue confidence in statistical estimates, leading to the under- or overestimations of disease presence, burden and spread. Underestimation may lead to an insufficient response, while overestimation can lead to focusing disease mitigation efforts too much on certain locations at the detriment of others. The opposite is also possible, where wildlife managers assume their small datasets are not reliable or do not push for surveillance efforts that are not expected to return high sample sizes, when in fact any surveillance data is better than no surveillance data, even a sample size of 1. The aim of our paper was thus to use real surveillance data from two CWD-affected U.S. states – Illinois and Wisconsin – to clarify the role of sample size in CWD apparent prevalence estimation and provide guidelines about the language to use when discussing statistics derived from surveillance data with limited testing.

To examine the relationship between AP and tests in Illinois and Wisconsin, we selected townships as the spatial scale for aggregating and evaluating CWD testing data because townships are the smallest area of land (mean = 83 km^2^) designated by the U.S. Public Land Survey System (USGS) wherein CWD surveillance data is publicly reported. The CWD apparent prevalence was calculated for each township and year (FY 2003 and 2022) with data and the summary statistics of these apparent prevalences are provided in Table 1, grouped by state (Illinois and Wisconsin).

**Table 1.** Summary statistics of chronic wasting disease (CWD) surveillance in Illinois and Wisconsin, USA, at the township level, between fiscal years 2003 and 2022 for all townships that tested at least 1 deer for CWD. It accounts for the number of animals tested (CWD tests) and CWD-positive animals (CWD cases). A fiscal year is the time between 1 July of one calendar year and 30 June of the following calendar year.

State	Covariate	Min	Q1	Median	Mean	Q3	Max
Illinois	CWD Tests	1	3	11	20.68	27	425
	CWD Cases	0	0	0	0.30	0	15
	Apparent Prevalence	0	0	0	0.01	0	1
Wisconsin	CWD Tests	1	4	11	30.06	30	1370
	CWD Cases	0	0	0	1.66	1	61
	Apparent Prevalence	0	0	0	0.04	0.02	1

Townships in Illinois and Wisconsin tested a similar number of deer for CWD, with Q1, median, mean, and Q3 values close in size. The maximum AP for both states was 100%, which is either alarming or indicative that something beyond the disease burden is at play. These states differed in their mean apparent prevalence, with the average Wisconsin township having six times more cases than the average township in Illinois. The third quartile for CWD cases in Wisconsin is 1, showing that the higher mean is not due to a few extreme values but to more widespread CWD (Table 1). This can be observed visually in the map of cumulative CWD cases in wild deer (FY2003–2022) in all affected Illinois and Wisconsin townships (Figure 1b,c). Figure 1a shows the total number of deer tested for CWD in this time period. Comparison between Figure 1a and b reveals that the majority of deer tested were from known CWD-positive townships.

**Figure 1.**
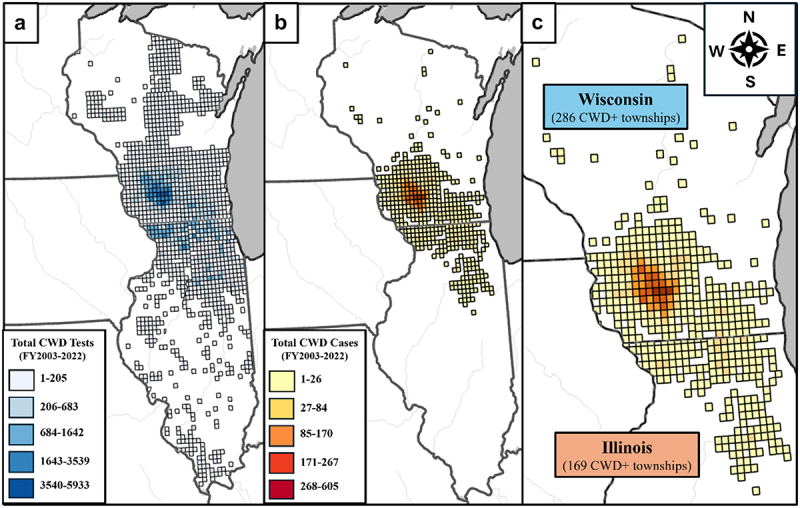
Map of all townships in Wisconsin and Illinois, USA, affected by chronic wasting disease (CWD). The cumulative number of white-tailed deer (*Odocoileus virginianus*) tested for CWD in each township between fiscal years 2003 and 2022 are shown in Figure 1a,1,1 show the cumulative number of CWD cases in this time period. A fiscal year is the time between July 1^st^ of one calendar year and June 30^th^ of the following calendar year.

The amount of testing is critical for determining the reliability of statistics, with 30 samples often quoted as the minimum sample size needed. However, whether this holds for CWD surveillance have not yet been explored. The scatterplots in Figure 2 visualize the relationship between the number of deer tested for CWD and AP at the township level. It should be acknowledged that Figure 2 cannot distinguish between high apparent prevalences due to low test numbers and high APs from herds with high true prevalences.

**Figure 2.**
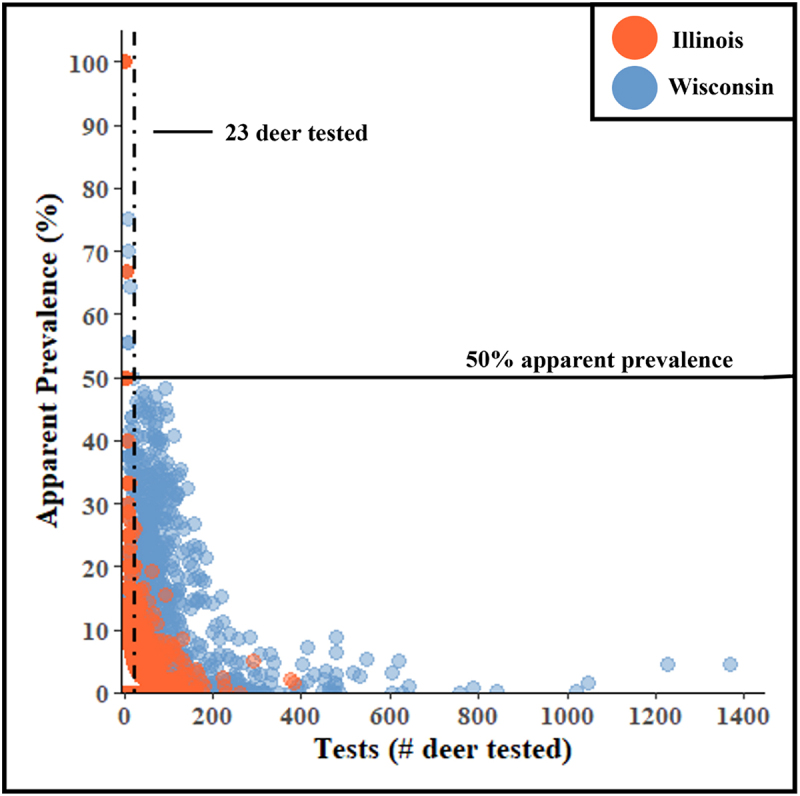
Scatterplots of the relationship between the number of white-tailed deer tested for chronic wasting disease in a township (x-axis) and the maximum apparent prevalence (AP) detected from each given number of tests (y-axis) in Illinois (orange) and Wisconsin (blue). Data points come from surveillance efforts in wild deer between fiscal years 2003 and 2023. The horizontal black line indicates 50% apparent prevalence, and the vertical dashed line identifies the minimum number of deer to test (23 deer) to avoid potentially inaccurate apparent prevalences ≥50%.

Figure 2 shows a negative relationship between AP and the number of animals tested when testing is limited and how this trend levels out as testing increases. Examination of Illinois and Wisconsin surveillance data identified 22 deer as the maximum number of tests that calculated an AP > 50%, making 23 deer a rough minimum threshold for testing to avoid APs that more reflect the sampling effort than the herd’s true prevalence.

Examination of the sources of extreme apparent prevalences revealed a total of 33 data points with an apparent prevalence >50%. These came from townships throughout the CWD-affected areas of Wisconsin and Illinois. Analyses of these data points show that 81% originated from townships with ≤2 deer tested for CWD cumulatively between FY 2003 and 2022. Of the remaining six observations, three came from townships with four or five tests and three observations came from townships with 6, 7 and 9 cumulative tests. These numbers support the general observation that high apparent prevalences come from townships with low testing.

Next, multilevel Bayesian zero-inflated regression was employed to better understand these dynamics, with Illinois and Wisconsin modelled separately to allow comparison between the states. Beta regression with a ‘logit’ link function was chosen because the apparent prevalence is a proportion bounded by 0 and 1. The Beta regression chosen was zero-inflated because 86% of Illinois and 73% of Wisconsin APs were 0’s and it was suspected that the processes that impact detecting CWD where it is present differ from those that yield AP’s of 0, which could arise either from insufficient surveillance in CWD-affected areas or surveillance in areas where CWD is absent. The unnested levels included in the models were ‘fiscal year’ and ‘township’ to account for similarities in AP attributable to underlying spatiotemporal relationships in the data. The ‘brms’ package [19–21] in R Core Team [22] was used for the modelling with 10,000 net iterations.

Different combinations of linear and nonlinear covariates – CWD cases and CWD tests – were tested to determine which model best fit the data. Fit was quantified by the leave-one-out cross-validation information criterion (LOOIC), with the smallest LOOIC indicating the best fit [23]. All models contained the linear terms ‘Tests’ and ‘Cases’ to account for the components of the AP equation. Nonlinear transformations of Tests and Cases were also included because the curves in Figure 2 suggest the presence of nonlinearity. Inverse, square root and log transformations were applied to the Tests covariate, while only the square root transformation was applied to the CWD cases covariate because the other transformations would necessitate removing all 0’s, which made up a high percentage of values for CWD cases (Illinois = 38%; Wisconsin = 72%). All combinations of covariates were explored. The Rhat statistic was used to determine model convergence, with a Rhat close to 1 indicating convergence [19–21]. The minimum effective sample size ratio – which should be >0.1 – was used to ensure that the data was sufficient for accurate results [19–21]. The Bayesian R^2^ was calculated to quantify the fraction of variance in the data each model explained and the LOOIC was used to compare models and identify the best fit.

For both Illinois and Wisconsin, the best fit model included the linear and inverse Tests covariates, and the linear and square root transformed Cases covariates. Table 2 reports the regression coefficients, relative ranks and 95% credibility intervals – the range that contains the true value of the parameter with a 95% probability [24] - for the best fit models. Relative ranks are based on the magnitude of the regression coefficient, with the largest magnitude corresponding to the most important covariate. A regression coefficient was statistically significant if the 95% CI did not include 0 [24].

**Table 2.** Results of the best-fit Bayesian zero-inflated Beta regression models quantifying the relationship between apparent prevalence and the number of chronic wasting disease cases (CWD cases) and deer tested for CWD (CWD tests) in township-ranges for Illinois and Wisconsin, USA. Statistically significant 95% credibility intervals (95% CI) are those that do not contain 0. Model coefficients cannot be compared between the separate models for Illinois and Wisconsin because these models are based on different datasets. Nonlinear transformations include the inverse transformation of tests (Tests1) and the square root transformation of cases (Cases0.5).

State	Covariate	Relative Rank	Regression Coefficient	95% CI
Illinois	Intercept	-	−5.11	[−5.32, −4.89]
	Tests1	1	8.11	[7.90, 8.32]
	Cases0.5	2	2.45	[2.20, 2.70]
	Cases	3	−0.34	[−0.41, −0.27]
	Tests	4	−0.02	[−0.02, −0.01]
Wisconsin	Intercept	-	−4.34	[−4.44, −4.23]
	Tests1	1	7.90	[7.71, 8.09]
	Cases0.5	2	1.42	[1.36, 1.48]
	Cases	3	−0.11	[−0.12, −0.01]
	Tests	4	−0.01	[−0.01, −0.01]

Since the models that examined Illinois and Wisconsin data are based on different datasets, their regression coefficients cannot be compared directly, but general observations can be made. First, the covariates included in the best fit models for both states were identical – Tests, Cases, Tests1, and Cases0.5 – and shared the same relative importance rank (Tests1 > Cases0.5 > Cases > Tests) (Table 2). This suggests that the relationships between CWD apparent prevalence, tests, and cases may not be state-specific, but an expanded analysis with other U.S. states is needed to evaluate that possibility. The ranking of the nonlinear covariates above the linear covariates also establishes that nonlinearity drives the relationship between apparent prevalence and its component variables, an important insight for future models. The Bayesian R^2^ of the Illinois’ best fit model was 27.2%, while this metric for Wisconsin was 30%, another similarity that shows only about 1/3 of data variance is accounted for in these models and other covariates exist that influence AP as well.

These regression coefficients also show that while the linear Tests and Cases have a significant negative relationship with AP, this relationship is significantly positive for Test1 and Cases0.5. This has critical management implications; when testing numbers are very low (< 23 deer), adding even a single test can meaningfully decrease the AP and reduce the chance of unrepresentatively high AP values. However, this relationship is not sustained indefinitely, as indicated by the large Tests1 and Cases0.5 which quickly dominate their respective dynamics with AP. That is not to say that it is not worth adding tests once a certain threshold is met – maximizing the number of deer tested for CWD should always be a priority – but rather that the instability of AP at low testing numbers can be viewed as an argument to invest in more testing when initial surveillance efforts yield limited results.

One unexpected observation was the negative relationship between Cases and AP at low testing numbers. Usually the detection of CWD-positive deer increases the AP, or at least does not decrease it, but these models identify conditions when this assumption does not hold. This is likely due to the initial sensitivity of AP to tests so that adding cases still leads to a decrease in AP just because adding cases means adding tests as well, further emphasizing the need to improve testing numbers.

There are several takeaways from these analyses. First, we observed graphically that large APs (≥50%) occur only when few deer (<23 deer) are tested, indicating that these APs are caused by insufficient testing rather than high true prevalences (Figure 2). This was reinforced by models that quantified a significantly negative relationship between AP and the number of CWD tests when, and only when, testing numbers are low. This emphasizes the need for additional testing of animals and the importance of being careful when using statistics derived from limited sample sizes (< 23 deer), which includes reporting results as ‘X cases in Y tests’. It should be noted, however, that testing 23 or more deer is not a guarantee that the apparent prevalence accurately reflects the true prevalence. It also does not mean that surveillance efforts that fail to meet the 23 deer threshold are not worthwhile. Wildlife managers face many barriers to achieving testing numbers that satisfy these sampling thresholds, including limitations with funding and staffing, so it is important to emphasize that any surveillance – even 1 or 2 tests – is better than none. This threshold of 23 deer should be used more as a means of judging the appropriate language to use when discussing the surveillance data, rather than as a blunt instrument to strike down insights gained from smaller datasets.

To be confident that the AP reflects the true prevalence, a full assessment of the sample sizes and uncertainty involved needs to be conducted. Thus, the insights gained from these analyses inform the use of CWD-related statistics but do not discount the potential for a significant disease presence and resulting large apparent prevalence values. We merely encourage caution when utilizing such statistics to avoid inappropriate use of them in a way that can reduce the effectiveness of management decisions and accuracy of communications with stakeholders.

Though the data from this study come from only two U.S. states, the negative relationship between sample size and AP at small sample sizes should hold regardless of the data source, though the threshold for omitting AP’s >50% may vary. The thresholds discussed herein should be roughly the same for other locations as long as cervids are primarily sampled via annual recreational hunter harvest in CWD-affected areas from herds continually exposed to the disease. This does pose a challenge when comparing to U.S. states or countries that employ different sampling strategies. The use of targeted sampling schemes based on deer demographics, geography, or other factors may result in smaller sample sizes being able to accurately detect the apparent prevalence. In contrast, focusing surveillance on areas around CWD-affected locations instead of within may require more than 23 deer to properly approximate the true prevalence because of the lower likelihood of finding CWD-positive animals. These limitations to our analysis highlight further opportunities to evaluate the role of sample size on apparent prevalence estimation in CWD surveillance systems [25–29].

## Data Availability

Data for cases of chronic wasting disease (CWD) in Illinois at the county level, as well as the number of deer tested for CWD, can be obtained from the Illinois Department of Natural Resources’ CWD Annual Reports (Illinois Department of Natural Resources; https://huntillinois.org/cwd-sampling/#cwd-annual-reports).
